# Effect of synthesis method on ammonium sorption behavior of oat husk biochar

**DOI:** 10.1038/s41598-025-89335-z

**Published:** 2025-04-01

**Authors:** José Daniel da Silva Fonseca, Karolina Matej-Łukowicz, Jacek Kluska, Mateusz Adam Baluk, Joanna Kulesza, Bráulio Silva Barros, Ewa Wojciechowska

**Affiliations:** 1 Programa de Pós-graduação em Ciência de Materiais, Centro de Ciências Exatas e da Natureza-CCEN, Universidade Federal de Pernambuco, Av. Prof. Morais Rego, 1235-Cidade Universitária, Recife, PE 50670-901 Brazil; 2 Faculty of Civil and Environmental Engineering, Gdansk University of Technology, Narutowicza 11/12, 80-233 Gdansk, Poland; 3 Institute of Fluid Flow Machinery, Polish Academy of Sciences, Fiszera 14, 80-231 Gdansk, Poland; 4 Department of Environmental Technology, Faculty of Chemistry, University of Gdańsk, Wita Stwosza 63, 80-308 Gdansk, Poland; 5 Departamento de Química Fundamental, Centro de Ciências Exatas e da Natureza-CCEN, Universidade Federal de Pernambuco, Av. Prof. Morais Rego, 1235-Cidade Universitária, Recife, PE 50670-901 Brazil; 6 Departamento de Engenharia Mecânica, Centro de Tecnologia e Geociências - CTG, Universidade Federal de Pernambuco, Av. Prof. Morais Rego, 1235- Cidade Universitária, Recife, PE 50670-901 Brazil

**Keywords:** Wastewater, Ammonium ion removal, Oat husk biochar, Adsorption, Modified biochar, Biochemistry, Environmental sciences

## Abstract

Ammonium is a pollutant commonly detected in various water sources and, in high concentrations, poses significant risks to environmental quality and public health. Numerous techniques have been developed and are currently employed to eliminate pollutants, including ammonium ions, from water. Among these various methods, adsorption has proven to be the most cost-effective and eco-friendly, particularly when utilizing an inexpensive and effective adsorbent. This study aims to determine the optimal method for producing biochar from oat husks, focusing on maximizing ammonium adsorption capacity among three selected preparation techniques. The chosen methods are based on a common combustion process and the use of chemical activators, such as ferrocene and sodium hydroxide. Biochar produced with sodium hydroxide exhibited the highest concentration of oxidized groups, as confirmed by Fourier transform infrared (FTIR) spectroscopy. The adsorption kinetics of ammonium via the sodium hydroxide-modified biochar followed the Avrami model. The adsorption isotherms were best described by the Freundlich (R^2^ > 0.9) and Langmuir (R^2^ > 0.8) models. The desorption behavior followed a first-order model. According to the Langmuir isotherm, the maximum adsorption capacity of the NaOH-modified biochar was 59.61 mg/g. The method utilizing sodium hydroxide was the most effective at preparing biochar with the highest adsorption capacity, indicating its potential applicability in the remediation of ammonium-contaminated water. This research also establishes a foundation for further investigation into methods for producing biochar with enhanced adsorption capabilities from the same agricultural waste source.

**Supplementary Information:**

The online version contains supplementary material available at 10.1038/s41598-025-89335-z.

## Introduction

Biochar has become known as a significant and widespread material owing to its numerous potential applications. Biochar is a porous, carbonaceous material produced through biomass pyrolysis, either in the presence or absence of oxygen. Biochar provides three primary benefits: it can be produced from waste materials, it is readily accessible, and its production costs are comparatively low.

A primary source of materials for biochar production is agricultural waste^1^. There are three principal categories of potential applications for biochar: using it for treating the environment, applying it to produce fertilizer, and employing it to produce energy. The first application is consistent with one of the UN goals of the United Nations (UN)^1^ - Goal 6: Clean water and sanitation. Ammonium nitrogen is one of the primary causes of water pollution^2^. Although the natural ammonium concentration in water sources is typically low, intensive human activities and industrial production have significantly increased ammonium runoff^3^. Ammonium contamination can severely impact various environments, including the soil, atmosphere, and rivers. The improper disposal of agricultural, industrial, and domestic waste can lead to significant economic and environmental issues^4,5^. The estimation is that more than 200 tons of NH_3_/NH_4_^+^ are produced annually worldwide^6^. The effective removal of NH_4_^+^ and mitigation of NH_3_ emissions from various sources are necessary to maintain water resources and reduce air pollution, which contributes positively to the environment and the economy. According to Gillingham et al.^7^. Xu et al.^8^, Huang et al.^9^ and Han et al.^10^ biochar can effectively absorb ammonium nitrogen and another water and wastewater pollution^11–13^. Adsorption-based methods exhibit high efficiency (> 90%) in removing pollutants while preserving the taste and color of water^14,15^.

Several types of production techniques should be checked in order to produce biochar with the most effective sorption parameters. The primary thermal decomposition methods employed in biochar production include pyrolysis, hydrothermal carbonization, gasification, and microwave heating, with synthesis parameters such as duration and temperature varying accordingly^16^. During biochar production, specific chemicals can be used to improve biochar characteristics, such as enhancing the formation of surface chemical groups and increasing surface area.

Yang et al.^17^. reported on biochar derived from the pyrolysis of pine sawdust at 300 °C for ammonium removal. Their research demonstrated that biochar prepared at relatively low temperatures presented a relatively high ammonium adsorption capacity, attributed to chemical bonding and electrostatic interaction sorption mechanisms. Vu et al.^9^. synthesized biochar from corncob and applied it to remove ammonium from wastewater in the concentration range of 10–100 mg/L. Their study included a modification process in which the biochar was soaked in a nitric acid solution and then in NaOH (with different molar concentration of both chemical), which increased its sorption capacity. There are numerous applications for biochar, thus the production process should be analyzed and improved depending on the biochar’s intended function.

Biochar’s adsorption mechanism for removing organic and inorganic pollutants may include electrostatic interaction, ion exchange, pore filling, and precipitation^10^.

The research study presented is the first to propose and compare numerous biochar production methods for one substance. The main objective of the research was to analyze three different processes to produce biochar from oat husks, a waste product that is highly available. The second objective was to identify the most effective process to produce biochar with a significant capacity for ammonium nitrogen sorption.

The findings of this study are expected to contribute significantly to the development of sustainable and efficient adsorbents for environmental remediation and, at the same time, can help to recover agro-waste as a value-added product.

## Materials and methods

### Materials

Among all the agro-waste used for biochar production, oat husk and straw have gained special attention due to Poland’s high grain production. Oat (*Avena sativa* L.) is a cereal from the *Poaceae* family. It grows well in marginal locations because of its minimal soil needs and tolerance to soil acidity. Poland is one of the largest oat producers in the world. Oat grain is used for feed and to produce gluten-free flour (oat flour), groats (oat groats) and flakes (oat flakes, oat cocoa, bran). According to the Food and Agriculture Organization of the United Nations (FAO), in 2022, Poland produced more than 1.5 billion tons of oats in 466,270 ha^18^. This valuable agricultural waste - oat husk- can be used as raw material for multifunctional biochar^19,20^.

### Biochar production

Biochar was produced using all three processes at a low temperature of 300 °C to achieve the greatest number of functional groups possible, which is particularly crucial for adsorption21.Method I: OAT-F.

The synthesis method for OAT-F was adapted from Somanathan et al.^21^. , who utilized this technique to produce graphene-like carbonaceous material from sugarcane bagasse^21^.The oat husks were crushed and ground into a fine powder. Approximately 2.5 g of this powder was mixed with 0.5 g of ferrocene, and the mixture was placed in a crucible and heated in a muffle furnace at 300 °C for 10 min under atmospheric conditions. The material was subsequently covered to prevent oxygen contact and cooled to ambient temperature. The resulting material was ground and soaked in 100 ml of a 5% HCl solution for 24 h. Afterward, the material was washed multiple times until a neutral pH was achieved. Finally, the washed biochar was dried at 60 °C overnight.

#### Method II: OAT-1 H

A crucible with a capacity of 3 L was placed inside the heating chamber. The final temperature was set to 300 °C and maintained for 1 h, with the temperature measured at the center of the crucible. The experimental setup was equipped with a PID controller to ensure precise temperature control, which activated the heater whenever the temperature in the heating chamber dropped below 300 °C. The resulting material was then ground into a fine powder. As described in Method I, the acid-washing procedure was subsequently repeated.

#### Method III: OAT-NAOH

The preparation method for the OAT-NAOH sample was adapted from Liu et al.^22^. , with modifications^22^. The oat husks were ground into a fine powder and then treated with a 1.0 M sodium hydroxide solution at a solid-liquid ratio of 1.0 g to 2.5 ml for 2 h. The alkali-treated material was subsequently oven-dried at 60 °C for 24 h. The dried material was then placed in a crucible, heated to a final temperature of 300 °C, and maintained for 2 h under a nitrogen atmosphere. The resulting biochar was ground into a fine powder and acid-washed following the procedure described in Method I.

### Biochar characterization

FTIR analyses were conducted using a Nicolet IS10 spectrometer in attenuated total reflectance (ATR) mode with a germanium crystal. Elemental analysis was performed using a CHNS/O Analyzer (Thermo Scientific). The elemental composition analysis was conducted using XRF methodology^23^. This method involves measuring the emission of characteristic fluorescent X-rays from a sample excited by X-ray irradiation. A Bruker Scientific Instruments device was used for this analysis. The morphology of the particles was examined via a JEOL JSM-7610 F scanning electron microscope (SEM) operating at 5 kV within a high vacuum environment.

### Initial ammonium sorption evaluation

The initial ammonium sorption capacity of each biochar sample was assessed via a modified version of the method described by Gao et al.^24^. A stock solution of NH_4_^+^-N with a concentration of 0.5 g/L was prepared by accurately dissolving 1.91 g of NH_4_Cl (analytical grade) in 1 L of ultrapure water. This stock solution was used for subsequent tests. To evaluate the initial sorption capacity of each biochar mixture for ammonium, approximately 0.1 g of biochar was mixed with 50 mL of the stock solution in a sealed 100 mL glass container. The containers were placed in a mechanical shaker set at 25 °C and agitated at 120 r/min for 24 h. The mixtures were subsequently filtered through 0.45 μm membrane filters. The ammonium concentration in the supernatants was determined via a UV/VIS spectrophotometer (the colourimetric indophenol blue method).

### Sorption isotherms

Based on the results from the initial sorption evaluation, the OAT-NAOH biochar was selected for further isotherm and kinetic experiments because it had the highest ammonium sorption capacity. For the sorption isotherm study, 0.1 g of biochar was placed in a sealed glass vessel and mixed with 50 mL of ammonium solution at various initial concentrations (50, 75, 100, 150, 200, and 500 mg/L). The mixtures were mechanically shaken at 120 r/min and maintained at 25 °C for 24 h. The mixtures were filtered through 0.45 μm membrane filters, and the ammonium concentration in the supernatants was subsequently determined via a UV/VIS spectrophotometer.

### Adsorption kinetics

For the adsorption kinetics study, 0.1 g of the biochar was placed in a sealed vessel with 50 ml of a 100 mg/L ammonium solution. The vessels were mechanically shaken at 120 r/min and maintained at 25 °C. Samples were taken at 15-minute intervals over a total duration of 2 h. of the experiments. After each interval, the mixtures were filtered through 0.45 μm membrane filters, and the ammonium concentration in the supernatants was measured via a UV/VIS spectrophotometer.

### Desorption kinetics

The desorption behavior of the biochar was evaluated by first investigating its adsorption capacity. The maximum adsorption capacity was reached after 2 h, and was assessed at this point, where it began to decrease. In a typical experiment, 0.1 g of biochar was added to a 250 ml sealed glass vessel containing 50 ml of a 100 mg/L ammonium solution. The samples were shaken mechanically at 120 r/min and maintained at 25 °C, with analyses conducted at intervals of 2, 4, 6, 20, and 24 h. Following each interval, the mixtures were filtered through 0.45 μm membrane filters, and the ammonium concentration in the supernatants was determined via a UV/VIS spectrophotometer.

### Mathematical models

The ammonium sorption capacity of the biochar was calculated via the following equation:

1 $$\:{Q}_{e}=\:\frac{\left({C}_{0}-{C}_{e}\right)\times\:V}{m}$$

where *Q*_*e*_ is the amount of ammonium adsorbed per unit weight of biochar (mg/g) at equilibrium, *C*_*0*_ is the initial ammonium concentration (mg/L), *C*_*e*_ is the ammonium concentration at equilibrium, *V* is the volume of the ammonium solution (L), and *m* is the mass (g) of the biochar.

The adsorption isotherms were modeled via the Langmuir and Freundlich equations. The Langmuir equation is given by:

2 $$\:{Q}_{e}=\:\frac{{C}_{e}{Q}_{m}}{\left({K}_{L}\:{+\:C}_{e}\right)}$$

where *Q*_*m*_ (mg/g) is the maximum adsorption capacity; *Q*_*e*_ (mg/g) is the adsorption capacity at equilibrium; *C*_*e*_ (mg/L) is the ammonium concentration at equilibrium; and *K*_*L*_ is the Langmuir constant.

The Freundlich equation is expressed as:

3 $$\:{Q}_{e}=\:{K}_{F}{C}_{e}^{1/n}$$

where *C*_*e*_ (mg/L) is the ammonium concentration at equilibrium, *K*_*F*_ is the Freundlich constant, and *1/n* (with *0 < 1/n < 1)* indicates that the sorption process is favorable.

The kinetic behavior of ammonium sorption was described via four kinetic models: pseudo-first-order^25^, pseudo-second-order^9^, Elovich^24^, and Avrami^26^. The equations for these models are as follows:

Pseudo-first order:

4 $$\:{q}_{t}=\:{q}_{e}(1-{e}^{-{K}_{1}t})$$

Pseudo-second order:

5 $$\:{q}_{t}=\frac{{K}_{2}{q}_{e}^{2}t}{1+{K}_{2}{q}_{e}t}$$

Elovich model:

6 $$\:{q}_{t}=\frac{1}{\beta\:}\:\text{l}\text{n}(\alpha\:\beta\:t+1)$$

Avrami model:

7 $$\:{q}_{t}={q}_{e}(1-{e}^{\left(-{\left({K}_{A}t\right)}^{{n}_{A}}\right)})$$

where *q*_*t*_ is the adsorption capacity (mg/g) at time *t*; *q*_*e*_ is the adsorption capacity at equilibrium (mg/g); *K*_*1*_, *K*_*2*_, and *K*_*A*_ are the rate constants for the pseudo-first-order, pseudo-second-order, and Avrami models, respectively; *β* is the adsorption constant (g/mg) in the Elovich model; and *N*_*A*_ is the Avrami time exponent.

For desorption modeling, the first-order model described by Karka et al.. was used^26^. The first-order desorption equation is given by:

8 $$\:{q}_{des}=\:{q}_{0}{e}^{-{k}_{des}t}$$

where *q*_*des*_ represents the maximum adsorption capacity at time *t* during desorption and where *k*_*des*_ is the desorption rate constant.

## Results and discussion

### Biochar characterization

#### Method of biochar preparation

Biochar characteristics such as specific surface area, pore size, type of functional groups, and structure strongly depend on the pyrolysis process. The pyrolysis temperature is especially important^27^. As the pyrolysis temperature increases, the carbon content, porosity, specific surface area and pH increase as well. However, increasing the temperature results in the loss of some of the functional groups, decreasing ion exchange and increasing energy consumption for the process^28,29^. Biochar intended for adsorption should have as many functional groups as possible, and the specific surface area or pore size are of secondary importance^30^.

In the first method of biochar preparation, ferrocene was used as a catalyst because of its ability to enhance the biomass oxidation process through interaction with the material. Ferrocene is a potent oxidizing agent employed in various applications, including catalysis, nanomedicine, supramolecular chemistry, and sensing^31^.

During the carbonization process, ferrocene interacts with the biomass, catalyzing reactions with both solid and volatile compounds. Under certain conditions, ferrocenium ions may act as moderate Lewis acid catalysts, promoting the oxidation of carbonaceous compounds^32^. Ferrocene can undergo a reversible one-electron oxidation process, producing ferrocenium ions that facilitate electron catalysis^33^. Due to these characteristics, ferrocene is a powerful catalyst for various reactions, highlighting its potential use in biochar production.

The second method employed for biochar production is the conventional process of biomass carbonization. Typically, carbonization occurs in an oxygen-limited environment, preventing complete biomass combustion due to insufficient oxygen. This partial combustion generates the heat required for the carbonization process. In contrast to pyrolysis, which is generally conducted in an oxygen-free environment, carbonization allows the presence of some oxygen to facilitate the process. Carbonization yields tar, gases, and charcoal as its primary byproducts. While tar and gases can be further processed or utilized as fuels, the biochar produced can be incorporated into the soil to enhance its properties^34^.

The third method of biochar preparation involves an alkali-catalyzed activation process. Alkaline activation enhances the biochar surface area and increases the presence of oxygen-containing functional groups. Sodium hydroxide (NaOH) and potassium hydroxide (KOH) are among the most commonly used alkali-activating agents^35^. Biochar treated with NaOH has been shown to possess a relatively high adsorption capacity due to increased oxygen-containing functional groups and a relatively high percentage of graphitic carbon on the biochar surface^36^. Additionally, potassium hydroxide could increase the presence of specific organic and inorganic groups on the activated carbon surface^37^. Furthermore, using KOH has been found to increase the presence of specific organic and inorganic groups on the activated carbon surface. Consequently, alkali-based activation is a promising approach for improving functional biochar properties.

#### Elemental composition

Table 1 presents the elemental composition of the biochar samples before and after the acid-washing treatment. The potassium (K) content in the washed samples significantly decreased, indicating that washing with HCL solution effectively removes K, which is consistent with findings by Zhang and coworkers^38^. However, an increase in the contents of iron (Fe), silicon (Si), calcium (Ca), phosphorus (P), and sulfur (S) was observed, suggesting that while HCl effectively removes K, it does not remove these other elements^39^. The literature reports that deashing treatment exposes adsorption sites, improves the pore structure, ionizes oxygen functional groups, and facilitates introducing additional functional groups^38,39^. As a result of the deashing process, the adsorption capacity of biochar is increased^40^. Consequently, only acid-washed biochars were used for all the sorption experiments.

**Table 1:** Elemental composition of the biochar before and after acid (HCl) washing.

Sample	Element composition (wt% of samples)					
	K	Fe	Si	Ca	P	S
	Before washing					
OAT-F	35.5	18.9	15.1	12.9	11.3	2.9
OAT-1 H	45.0	2.0	18.1	14.9	12.5	2.6
OAT-2 H-NAOH	42.1	5.5	15.0	14.5	12.5	2.8
	After washing					
OAT-F	10.4	27.2	29.7	9.9	12.3	6.5
OAT-1 H	30.0	2.2	31.3	16.3	12.1	5.4
OAT-2 H-NAOH	1.1	2.8	59.4	12.8	N/D	8.4

The aromaticity of biochar is indicated by the H/C ratio^41^. The differences in H/C ratios were insignificant for the three synthesized biochars, suggesting that the synthesis method did not substantially affect the aromaticity and structural stability. A lower H/C ratio indicates greater aromaticity and a more stable biochar structure. The polarity and hydrophilicity of biochar are related to the O/C and (O + N)/C ratios; higher values indicate stronger polarity^42^. Increasing the temperature during synthesis enhances aromaticity but reduces polarity. Since all the biochars were synthesized at the same temperature, variations in the O/C and (O + N)/C ratios are attributed to the preparation methods. Compared with the other two samples, the OAT-2 H-NAOH sample presented a higher O/C ratio, suggesting that using NaOH increased the polarity of the biochar and, consequently, the number of oxygen-containing functional groups on its surface. The elemental composition of the synthesized biochars is presented in Table 2.

**Table 2:** Elemental composition of synthesized oat biochars.

Biochar	Component (%)				Atomic ratio		
	C	H	O^a^	*N*	H/C	O/C	(O + *N*)/C
OAT-F	63.72	4.67	29.79	1.82	0.07	0.47	0.50
OAT-1 H	60.00	4.72	33.68	1.60	0.08	0.56	0.59
OAT-2 H-NAOH	58.62	4.93	35.81	0.64	0.08	0.61	0.62

#### FTIR

According to Pantoja et al.^43^ feedstocks that create aromatic functional groups throughout pyrolysis are useful for ammonium removal. The analysis of the FTIR results primarily focused on functional groups containing oxygen, which facilitated the adsorption of ammonium^8^. The majority of these oxygen-containing functional groups possess a negative charge, enabling them to readily adsorb positively charged ammonium ions.

**Fig. 1:**
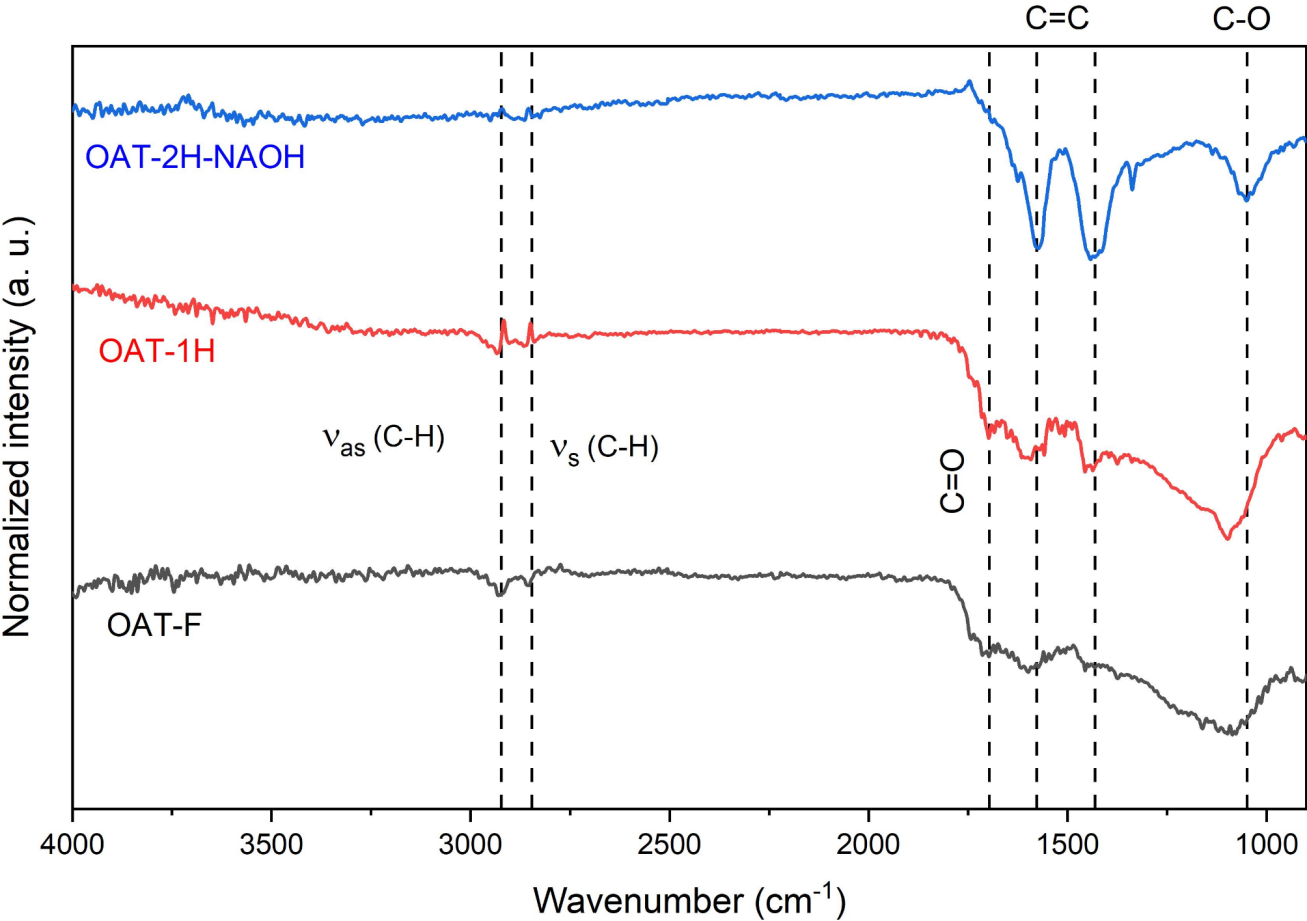
FTIR spectra of the synthesized biochar from OAT husk. OAT-F is the biochar synthesized utilizing ferrocene as a catalyst, OAT-1 H is the biochar produced by the standard carbonization procedure, and OAT-2 H-NAOH is the biochar produced with sodium hydroxide as a catalyst.

The 2923 cm^−1^ and 2852 cm^−1^ bands correspond to aliphatic groups’ asymmetric and symmetric C–H stretching vibrations. Carboxyl C = O stretching from carboxylic acids is observed at 1696 cm^−1^ for OAT-F and OAT-1 H with greater intensity than for OAT-2 H-NAOH. According to Zhang et al. (2013) C = O and –COO functional groups may react with ammonium ion. This reduced intensity in the sodium hydroxide-treated sample suggests that carboxylic acid groups are decomposed due to basic treatment. According to Vu^9^, the decrease in the carboxylic acid band can be attributed to the reaction between the O-H groups in carboxylic acids and NaOH during synthesis. Other notable bands include aromatic ring C = C vibrations at 1578 cm^−1^ and C–C stretches in aromatic rings at 1430 cm^−1^. The bands at 1052 cm^−1^, corresponding to C–O, C–C, and C–O–H vibrations in polysaccharides, can be attributed to the lignin, cellulose, and hemicellulose contents of the raw material used to prepare the biochar^44–46^. These bands shifted to approximately 1100 cm^−1^ in the OAT-1 H and OAT-F samples, and a shift was not observed in the NaOH-treated samples. Figure 1 shows the FTIR spectra^47^.

#### SEM and BET

**Fig. 2:**
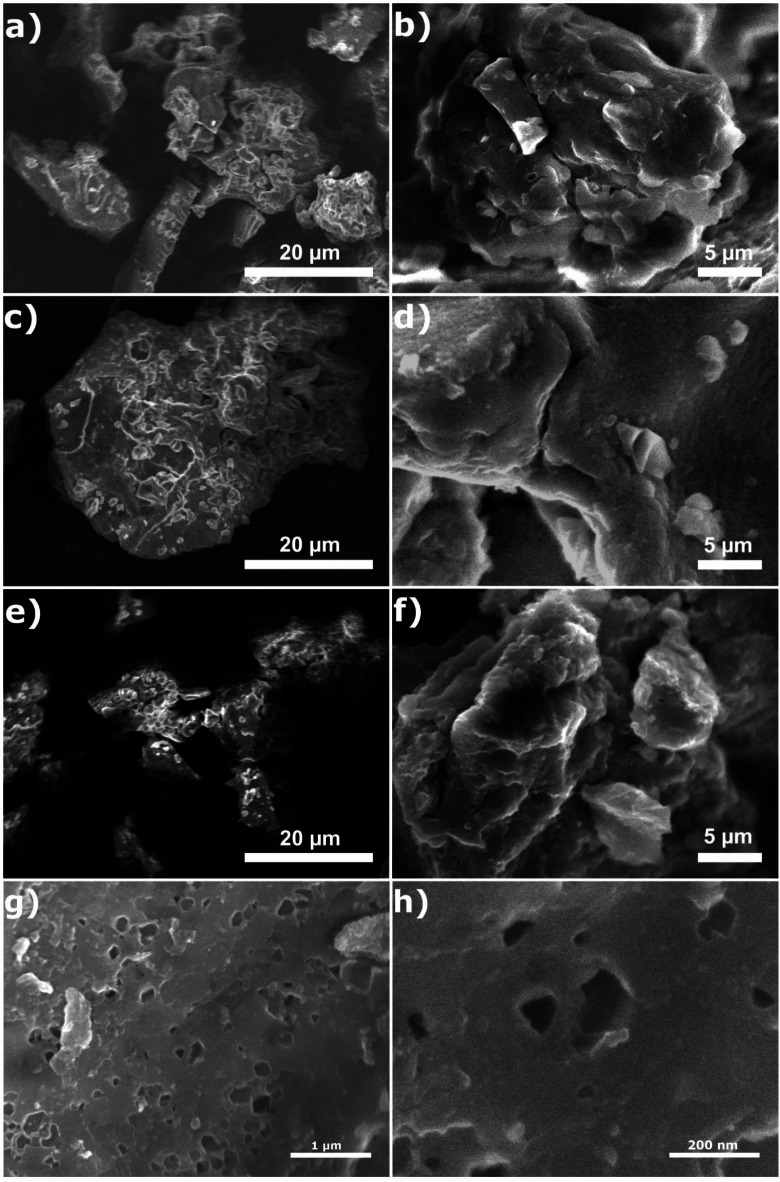
Morphology characteristics of the synthesized biochars. (**a**,**b**) OAT-1 H; (**c**,**d**) OAT-F; (**e**–**h**) OAT-2 H-NAOH; (**g**) The image was obtained using an acceleration voltage of 5 kV and a secondary electron detector. The magnification was 37,000x, and the scale bar represents 1 μm. (**h**) The image was obtained using an acceleration voltage of 5 kV and a secondary electron detector. The magnification was 140,000x, and the scale bar represents 200 nm.

SEM micrographs of the OAT-1 H, OAT-F, and OAT-2 H-NAOH biochars are presented in Fig. 2. The micrographs of OAT-1 H (Fig. 2a and b) reveal large agglomerates of particles, suggesting that the conventional carbonization process was insufficient to produce a biochar with tiny particles and macropores. The biochar produced with ferrocene, OAT-F (Fig. 2c and d), exhibited similar agglomerates to those found in OAT-1 H but with a more oxidized surface, as evidenced by the increased number of small particles on the biochar surface. This finding indicates that ferrocene can produce biochar with a rougher surface than conventional carbonization without chemical additives. These findings suggest that sodium hydroxide treatment can increase the roughness of the biochar surface. Additionally, micropores on the biochar surface indicate that using an alkaline base during synthesis can contribute to micropore formation. Particle size of three biochars has been shown in the charts in Appendix 1.

The results of BET surface area measurements indicate the highest value for OAT-2H-NAOH biochar (24.9 m^2^/g), where for OAT-1 H it was 1.9 m^2^/g, and for OAT-F it was 5.5 m^2^/g. The results of pore volume measurements were 3.87 × 10^−3^ for OAT-1 H, 1.20 × 10^−2^ cm^3^/g for OAT-F, and 3.28 × 10^−2^ for OAT-2H-NAOH. The largest pores were measured for OAT-1 H biochar (4.8 nm), while for OAT-F it was 3.8 nm, and for OAT-2H-NAOH 2.5 nm.

When comparing the three selected approaches, it is evident that the chemical activators’ methods resulted in a higher surface area than the conventional carbonization process. Additionally, biochars produced with chemical activation exhibited increased pore volume, corroborated by SEM micrographs showing a more brittle surface. Conversely, using chemical activators led to a reduction in pore size. Among the synthesized samples, OAT-2 H-NAOH (Fig. 2e and f) demonstrated the highest surface area and the smallest pore size. The Fig. 2g and h shows SEM micrographs for OAT-2 H-NAOH at different magnifications. The elevated surface area of the OAT-2 H-NAOH sample can be attributed to the increased oxidation caused by sodium hydroxide treatment^47,48^.

If we compare the BET results to those obtained by other Authors, the ones obtained are in the median. Some of the highest recorded were 470 and 448.2 m^2^/g lda for Bamboo in and Peanut shell, respectively^47,48^. However, these values were obtained for temperatures of 700 and 600 °C, respectively, and for the same materials at lower temperatures the values were < 10 m^2^/g. A more similar material may be buckwheat husk, the results of which were presented by Zama el al^49^, and for temperatures in the range of 350–650 °C, the values ranged from 10.7 to 17.8 m^2^/g, so it was lower than to OAT-2 H-NAOH.

### Initial ammonium sorption evaluation

Initial tests were conducted using a 100 mg/L ammonium solution to evaluate the ammonium sorption capacity of the biochars. All three biochars adequately removed ammonium from the aqueous solution. Among the samples, OAT-2 H-NAOH exhibited the highest ammonium sorption capacity at 7.9 mg/g. For comparison: OAT-1 H 1.1 mg/g, and OAT-F 5.1 mg/g. These findings suggest that base treatment with NaOH can increase the ammonium sorption capacity of biochar. Previous studies by Liu et al.. reported that alkali-modified biochars exhibit significantly high sorption capacities for ammonium removal from water^22^. Vu et al.^9^. after two-stage modification with KNO₃ and then with NaOH, they obtained higher results (22.6 mg/g), but they used more reagents.

The OAT-2 H-NAOH sample was selected for further kinetic and sorption isotherm studies, given its superior sorption capacity.

### Effect of contact time

**Fig. 3:**
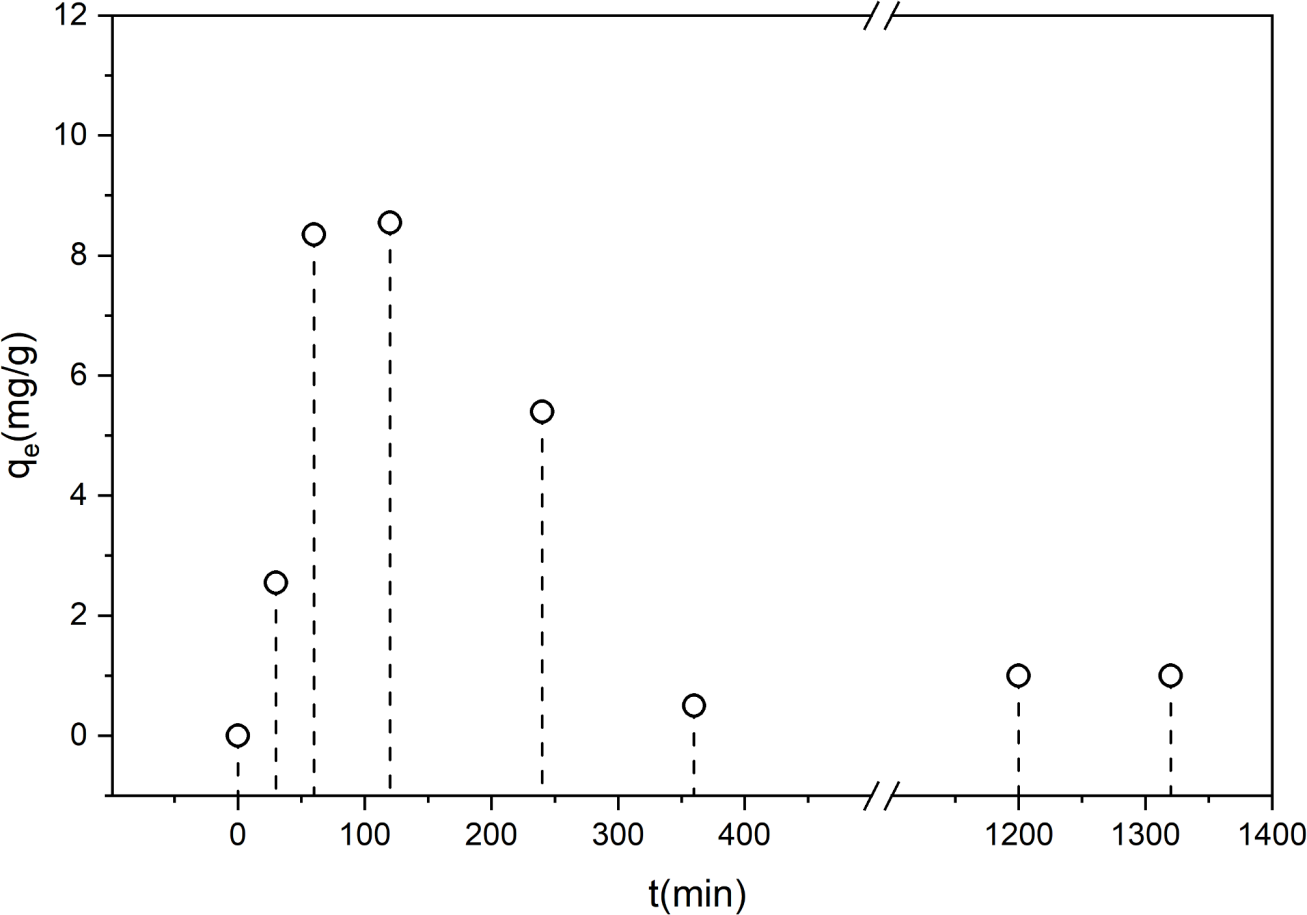
Effect of contact time on ammonium adsorption by OAT-NAOH over a 24 h period in a 100 mg/L ammonium solution.

The adsorption capacity of the OAT-2 H-NAOH sample is unusual. Figure 3 shows the effect of contact time on ammonium adsorption by the OAT-2 H-NAOH sample. An initial increase in adsorption was observed after 30 min, followed by a significant increase at approximately 1 h into the experiment. The maximum adsorption capacity is achieved after 2 h and then begins to decrease, reaching the lowest level after 24 h. This pattern suggests that OAT-2 H-NAOH reaches its saturation point for ammonium adsorption within 2 h of contact. Beyond this period, the adsorption capacity decreases. That results are similar to ammonium adsorption of LECA (Light expanded clay aggregate) – 2 h, which is used as a popular sorbent of selected pollutants from water^50^ or by almond kernel biochar − 1 h^51^.

### Adsorption kinetics

**Fig. 4:**
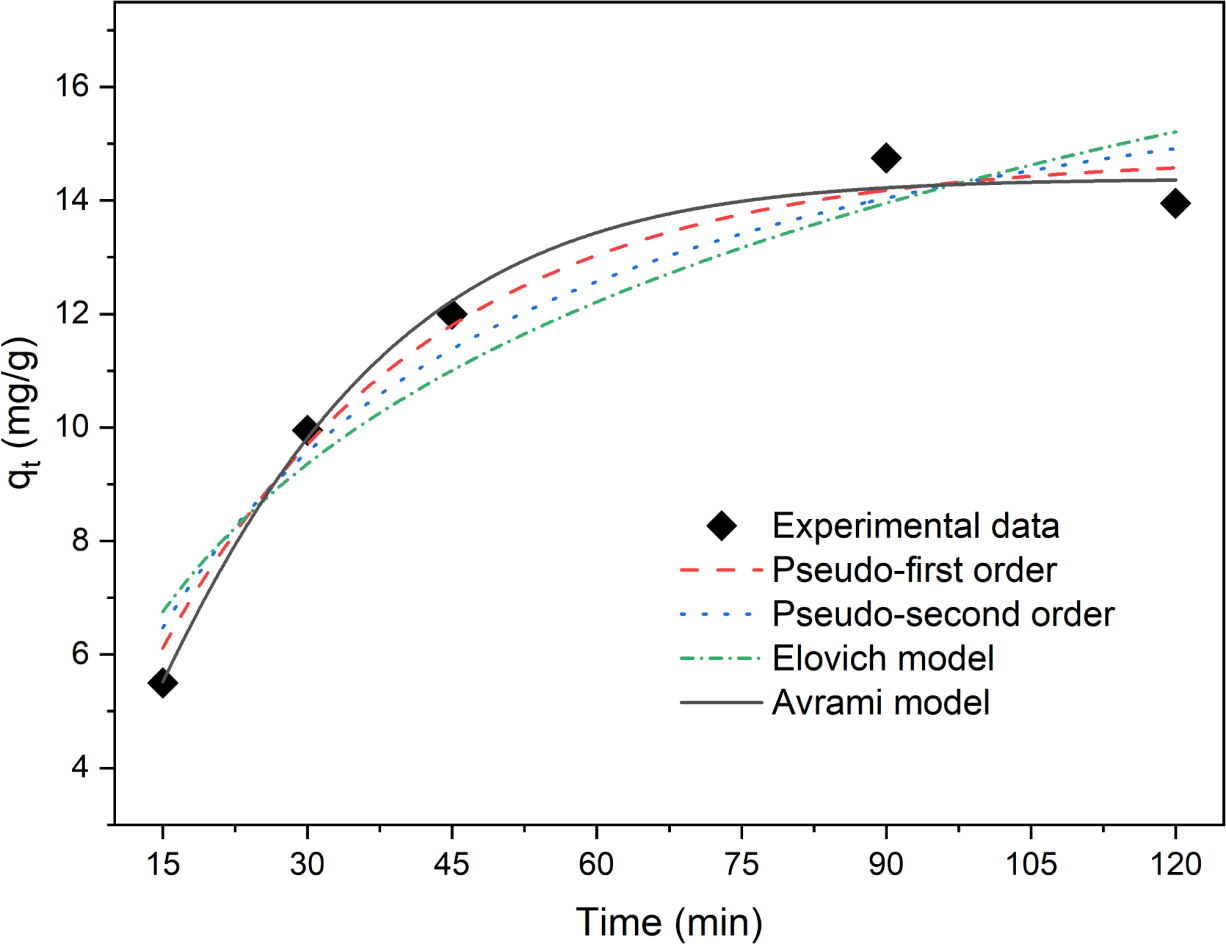
Experimental data and predicted kinetics models for ammonium sorption on OAT-2 H-NAOH. The figure illustrates the kinetics of ammonium sorption, complemented by parameters from the best-fit kinetic models. The following values were obtained for the various models: Pseudofirst Order (q_e_ = 14.780, K_1_ = 0.036, R^2^ = 0.978), Pseudosecond Order (q_e_ = 18.328, K_2_ = 0.0019, R^2^ = 0.947), Avrami (q_e_ = 14.383, K_av_ = 0.0372, N_av_ = 1.24, R^2^ = 0.990), and Elovich (α = 1.033, β = 0.220, R^2^ = 0.906). These parameters provide insights into the kinetics of ammonium sorption and illustrate the model fit to the experimental data.

Given that the maximum adsorption capacity was reached after 2 h, kinetics experiments were conducted within this period. The experimental data and corresponding kinetic models are depicted in Fig. 4. The simulations using the pseudo-first-order, pseudo-second-order, Avrami, and Elovich models all adequately described the data, achieving *R*^2^ values higher than 0.90. Among these models, the Avrami model provided the best fit to the experimental data, with an *R*^2^ value of 0.99 and a *q*_*e*_ of 14.4 mg/g. The fitting of the experimental data to the Avrami model suggests that the adsorption mechanism of ammonium on OAT-2 H-NAOH is governed by a complex multipath adsorption process^52^. Multiple adsorption pathways, such as surface diffusion, intraparticle diffusion, and physical and chemical interactions between CO_2_ and the active sites, are taken into account by Avrami’s fractional kinetic model^53^. The Avrami model was created initially to simulate phase transitions and crystal growth, but it has recently been used to explain CO_2_ adsorption onto surfaces that have been functionalized with amines, and then to ammonium adsorption processes^54,55^.

### Desorption kinetics

**Fig. 5:**
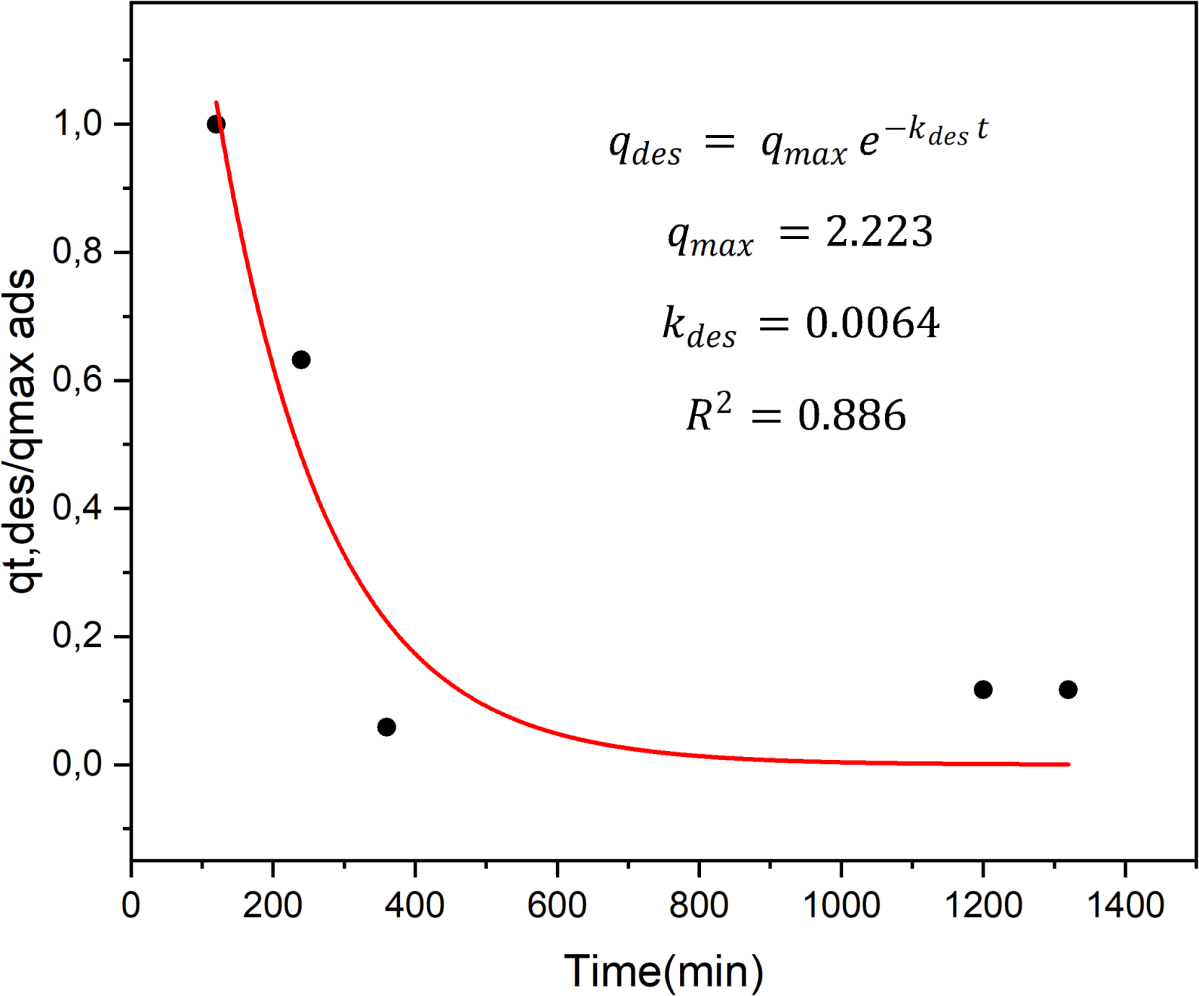
Desorption kinetic behavior predicted by the first-order model (red line) of OAT-2 H-NAOH (black dots).

Figure 5 shows the ammonium desorption behavior of the OAT-2 H-NAOH biochar, which was fit with a first-order model. The *R*^2^ value of 0.886 indicates a reasonable fit, suggesting that the first-order model satisfactorily describes the ammonium desorption performance of the OAT-2 H-NAOH biochar.

### Sorption Isotherm

**Fig. 6:**
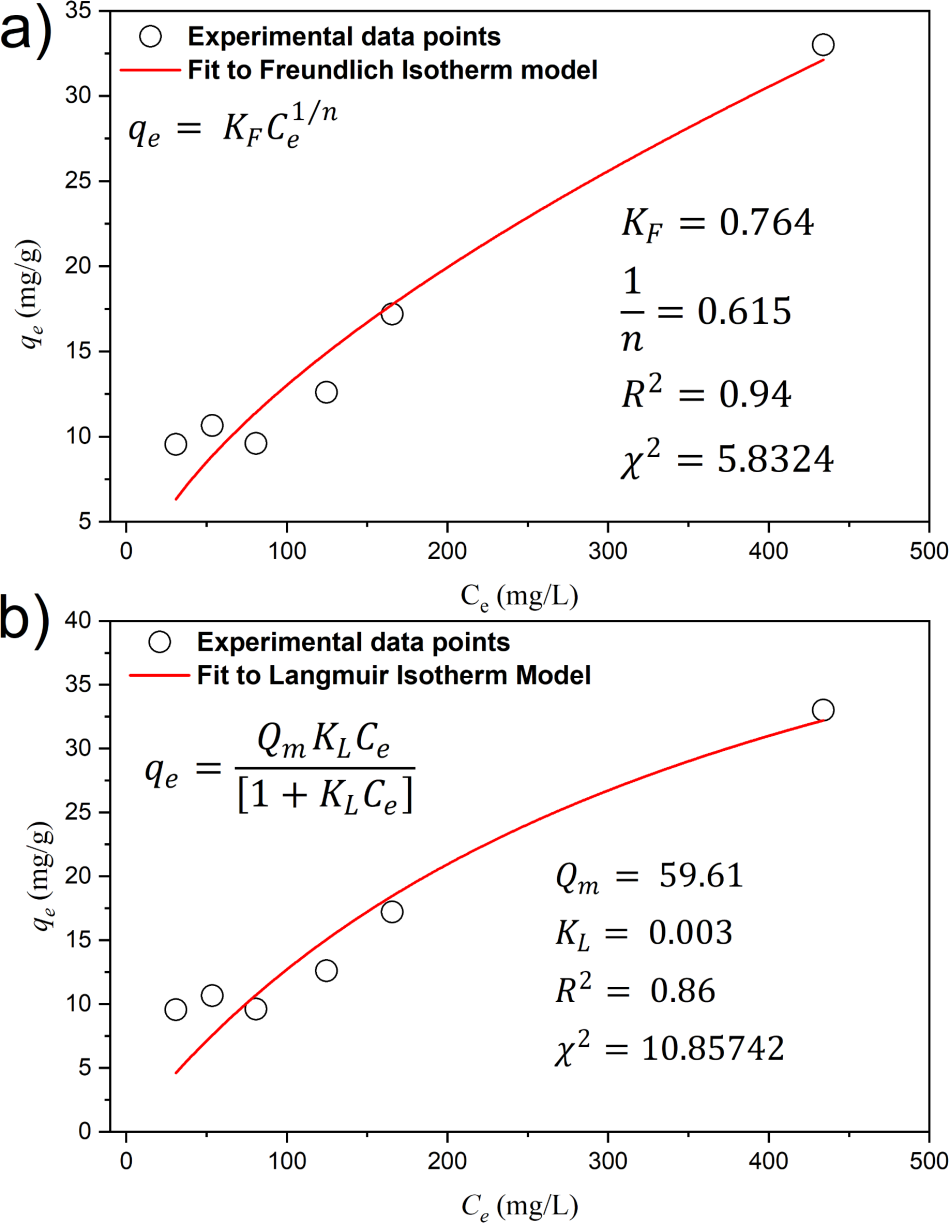
Ammonium adsorption prediction on OAT-2 H-NAOH via the Freundlich (**a**) and Langmuir (**b**) model.

The Freundlich and Langmuir models were used to elucidate the sorption isotherms of the OAT-2 H-NAOH biochar. The Freundlich model, which is applied to describe nonlinear adsorption phenomena^56^, involves empirical parameters such as *K*_*F*_ (L^1/n^.mg^1-1/n^.g^-1^) and *n*, where *q*_*e*_ represents the amount of adsorbate per unit mass of solid and *C*_*e*_ represents the equilibrium concentration of the adsorbate in solution^57^. Notably, favorable adsorption occurs when 0 < 1/*n* < 1. A graphical representation of the experimental data fitted to the Freundlich model is shown in Fig. 6.

Conversely, the Langmuir model is a prominent tool for characterizing gas-solid adsorption^58^. In this model, the parameter *K*_L_ governs interaction energies, whereas *Q*_*m*_ represents the Langmuir maximum adsorption capacity (mg/g). Figure 6 shows the overlay of experimental data for OAT-2 H-NAOH biochar with the Langmuir model.

When the fits of the Freundlich and Langmuir models were compared with the experimental data, the Freundlich model exhibited superior congruence, as evidenced by its higher regression coefficient (R^2^). This observation suggests that the multilayer adsorption process is more accurately captured by the Freundlich model. However, the Langmuir model demonstrated a satisfactory fit (R^2^ = 0.86), implying that the adsorption process of ammonium on the OAT-2H-NAOH biochar was influenced by multiple adsorption phenomena. According to the Langmuir fit, the maximum adsorption capacity of the OAT-2H-NAOH biochar was determined to be 59.61 mg/g.

It was also analyzed the ammonium removal capacities of biochars obtained from different sources (Table 3). When compared to commercial activated carbon^59^. Corncob^9^, pipeapple peel, sorghum straw or maple wood^60^, a commonly used sorbents, the biochar produced in this study demonstrates significantly higher adsorption capacity. Unlike peanut shell biochar^61^, which utilized ammonium sulfate as the ammonium source, the oat husk biochar from this study exhibited superior adsorption capacity relative to the other biochars. Among the twenty-two different biochars listed by Gilingham et al.^7^, OAT-2 H-NAOH biochar performed better than seventeen of them and worse only than only five: rice husk^62^, hardwood^63^, wood^62^, presscake^64^, and corn cobs^7^.

**Table 3:** Ammonium sorption capasity by different biochars.

Biochar	NH₄ sorption [mg/g]	Source
Commercial activated carbon	2.3	59
Corncob	22.6	^16^
Pipeapple peel	5.6	60
Sorghum straw	7.1	60
Maple wood	5.0	60
Peanut shell	123.2	61
Rice husk	71.94	62
Hardwood	114.2	^63^
Wood	133.33	62
Presscake	136.2	^64^
Corn cobs	243.3	^7^

## Conclusion

In this study, we determined that the biochar synthesized with sodium hydroxide presented the highest adsorption capacity compared with those produced via ferrocene and the standard carbonization process. The higher adsorption capacity of the sodium hydroxide-treated sample can be attributed to higher surface area and the presence of oxidized surface groups, as evidenced by BET and FTIR analysis, respectively. The biochar demonstrated maximum adsorption capacity within two hours, after which the adsorption capacity decreased, liberating the ammonium back to the solution and showing an unusual behavior for absorbents. This behavior indicates its suitability for rapid sorption/desorption applications. The data obtained from the kinetics and sorption experiments were best fitted to the Freundlich and Avrami models, respectively. We conclude that oat-husk biochars modified with sodium hydroxide are promising adsorbents for the removal of ammonium from wastewater.

## Electronic supplementary material

Below is the link to the electronic supplementary material.

Supplementary Material 1

## Data Availability

The data has been deposited on the Gdańsk University of Technology platform and is available at: https://mostwiedzy.pl/pl/open-research-data/oat-husk-biochar-charteristics-and-sorption-experiment,10250849571057131-0 (doi: 10.34808/by3s-nx35).
