# Supplementary material for: Effect of synthesis method on ammonium sorption behavior of oat husk biochar
Source: Sci Rep. 2025 Apr 1;15:11143. doi: 10.1038/s41598-025-89335-z (PMC11961723; doi:10.1038/s41598-025-89335-z)
Supplement: Supplementary file 1 — Supplementary Material 1 [file 41598_2025_89335_MOESM1_ESM.docx]

Appendix 1 – Chart with biochar particle size for three biochars: OAT-1H,OAT -F, OAT-2H-NAOH

Figure 1. Particle size of biochar OAT-1H

Figure 2. Particle size of biochar OAT-F

Figure 3. Particle size of biochar OAT-2H-NAOH
